# A comprehensive analysis of SLC25A1 expression and its oncogenic role in pan-cancer

**DOI:** 10.1007/s12672-023-00830-z

**Published:** 2023-11-19

**Authors:** Xin You, Lingling Huang, Ouxiang Huang, Yujie Deng, Xi Shi

**Affiliations:** 1 Department of Oncology, The First Affiliated Hospital of Fujian Medical University, No. 20 Chazhong Road, Fuzhou, 350005 Fujian China; 2 Molecular Oncology Research Institute, The First Affiliated Hospital of Fujian Medical University, No. 20 Chazhong Road, Fuzhou, 350005 Fujian China; 3 Department of Oncology, National Regional Medical Center, Binhai Campus of the First Affiliated Hospital, Fujian Medical University, Fuzhou, 350212 China

**Keywords:** SLC25A1, Pan-cancer, Prognosis, Tumor immunity, Therapeutic target

## Abstract

**Objective:**

The solute carrier family 25 member 1 (SLC25A1) is currently the only known human transporter for citrate in the mitochondrial membrane. However, its role in cancer development remains to be elucidated. We aim to analyze the expression profile, prognostic value, potential immunological significance, and effect on tumor growth of SLC25A1 at a pan-cancer level.

**Methods:**

Herein, the role of SLC25A1 in tumorigenesis and progression was investigated based on the Cancer Genome Atlas (TCGA), Gene Expression Omnibus (GEO), Genotype-Tissue Expression (GTEx), Clinical Proteomic Tumor Analysis Consortium (CPTAC), GeneMANIA, STRING and Cancer Dependency Map Project (DepMap) database via online websites or the R software. The protein expression levels were validated in tissue microarrays, and the effects on tumor cell lines were accessed through MTS and colony formation assays.

**Results:**

The expression of SLC25A1 increased in most cancers, and the upregulation of SLC25A1 in colon adenocarcinoma and lung adenocarcinoma was further confirmed by immunohistochemistry. Meanwhile, *SLC25A1* was linked to clinical outcomes across multiple tumor types, particularly in lung adenocarcinoma, where its high expression predicted poor prognosis. Moreover, *SLC25A1* was positively associated with MSI, TMB, and *CD276* and tightly correlated with tumor-infiltrating immune cells. Furthermore, the knockout of *SLC25A1* demonstrated inhibitory effects in most cancer cell lines in the DepMap project. Cellular experiments showed that *SLC25A1* knockdown significantly reduced the proliferation of lung adenocarcinoma cells.

**Conclusions:**

Our findings suggest the potential of SLC25A1 as a prognostic biomarker for cancers and a therapeutic target for precise antitumor strategy and cancer immunotherapy.

**Supplementary Information:**

The online version contains supplementary material available at 10.1007/s12672-023-00830-z.

## Introduction

Cancer ranks as a leading cause of death worldwide, with almost 10.0 million cancer-related deaths estimated to have occurred in 2020 [1]. Over the past decades, although breakthroughs in novel therapy methods have been made, especially targeted therapy and immunotherapy, the clinical outcome of cancer patients remained unsatisfactory [2]. Thus, the identification of new prognostic biomarkers and therapeutic targets in cancers is urgently needed. In recent years, the research on cancer metabolism has been progressing rapidly. A number of studies have revealed that cancer cells rely on metabolic reprogramming to satisfy their biosynthetic and reduction–oxidation demands during tumorigenesis and tumor progression [3, 4]. In addition, evidence accumulating suggests that metabolic reprogramming in cancer cells might influence the therapeutic effects of various anti-cancer drugs, including immune checkpoint inhibitors (ICIs) [5]. Therefore, extensive efforts have been focused on the roles of key metabolic enzymes and transporters in cancer diagnosis and treatment.

The solute carrier family 25 member 1 (SLC25A1), originally known as citrate transporter protein (CTP) or mitochondrial citrate/isocitrate carrier (CIC), is encoded by the *SLC25A1* gene located on chromosome 22q11.21 [6]. It is the only identified human transporter for citrate in the mitochondrial membrane to date. The known function of SLC25A1 is to promote the efflux of citrate exchanged with malate from the mitochondria to the cytosol or to mediate the reverse transport of cytosolic citrate to the mitochondria [7]. Citrate provides the source of two-carbon acetyl-CoA for de novo fatty acid (FA) synthesis in the cytoplasm, while enters the tricarboxylic acid (TCA) cycle for oxidative phosphorylation (OXPHOS) in the mitochondria [8]. As such, SLC25A1 is an essential transporter that bridges mitochondrial metabolism and lipid metabolism. Abnormal expression or activity of SLC25A1 has been found to be involved in inflammation, cancer, and other diseases [7]. Of note, an increasing number of studies have indicated that SLC25A1 may play an oncogene-like role in the development of tumors [6, 9]. SLC25A1 is highly expressed in breast, lung, and colorectal cancer, where its expression is closely associated with advanced clinicopathological features and poor prognosis [10–12]. Moreover, SLC25A1 has been reported to be functionally involved in tumor stemness, radioresistance, and chemotherapy resistance by reprogramming energy metabolism, maintaining redox homeostasis, and supporting lipid biosynthesis [11, 13, 14]. Furthermore, genetic suppression or pharmacologic inhibition of SLC25A1 significantly inhibited tumor growth and metastasis in a variety of common human cancer cell lines, whereas SLC25A1 overexpression enhanced the malignant phenotype [7, 10, 15]. Overall, SLC25A1 is attracting growing attention as a potential prognostic marker and a promising therapeutic target in several tumors. However, the landscape of *SLC25A1* gene expression and its biological function in different types of human cancers remains elusive.

In this study, we systematically analyzed the SLC25A1 expression across various human tumors using public databases and immunohistochemistry. Meanwhile, the associations of SLC25A1 with prognostic value, molecular pathways, immune infiltration, and other immune-related biomarkers in pan-cancer were also explored based on online bioinformatics tools and R program. In addition, in vitro functional validation showed that SLC25A1 knockdown attenuated the growth rate of LUAD cells. The findings of this study offer a basis for understanding the roles and potential mechanisms of SLC25A1 in human tumors, and provide a novel perspective for the application of SLC25A1 in cancer therapy.

## Material and methods

### SLC25A1 expression analysis

The SLC25A1 expression data across different tissues and cells under physiological conditions were obtained from the Human Protein Atlas (HPA) dataset (https://www.proteinatlas.org/humanproteome/pathology) [16]. The genetic alteration of *SLC25A1* status in all TCGA tumors was performed by the cBioPortal web tool (https://www.cbioportal.org/) [17]. To compare the expression level of *SLC25A1* gene in normal and tumor tissues, gene expression profiles and clinical information of 33 tumors and normal tissue samples were obtained from the TCGA database [18] (https://portalgdc.cancer.gov/) and the GTEx database [19] (https://gtexportal.org/) combined. The abbreviations and full names of 33 tumors are shown in Table 1. Statistical analysis was performed using R software v4.0.3. The CPTAC database in the UALCAN tool (http://ualcan.path.uab.edu/index.html) was used to analyze the SLC25A1 protein expression between different cancer tissues and normal tissues [20]. *P* < 0.05 was considered to be significant.

**Table 1:** Abbreviations and full names of 33 tumors in TCGA database

Abbreviation	Full name
ACC	Adrenocortical carcinoma
BLCA	Bladder urothelial carcinoma
BRCA	Breast invasive carcinoma
CESC	Cervical squamous cell carcinoma
CHOL	Cholangiocarcinoma
COAD	Colon adenocarcinoma
DLBC	Diffuse large B cell lymphoma
ESCA	Esophageal carcinoma
GBM	Glioblastoma multiforme
HNSC	Head and neck squamous cell carcinoma
KICH	Kidney chromophobe
KIRC	Kidney renal clear cell carcinoma
KIRP	Kidney renal papillary cell carcinoma
LAML	Acute myeloid leukemia
LGG	Brain lower grade glioma
LIHC	Liver hepatocellular carcinoma
LUAD	Lung adenocarcinoma
LUSC	Lung squamous cell carcinoma
MESO	Mesothelioma
OV	Ovarian serous cystadenocarcinoma
PAAD	Pancreatic adenocarcinoma
PCPG	Pheochromocytoma and paraganglioma
PRAD	Prostate adenocarcinoma
READ	Rectum adenocarcinoma
SARC	Sarcoma
SKCM	Skin cutaneous melanoma
STAD	Stomach adenocarcinoma
TGCT	Testicular germ cell tumors
THCA	Thyroid carcinoma
THYM	Thymoma
UCEC	Uterine corpus endometrial carcinoma
UCS	Uterine carcinosarcoma
UVM	Uveal melanoma

### Tissue microarrays and immunohistochemistry (IHC)

The tissue microarrays (HOrgC120PG05, HLugA150CS03 and HColA150CS02) were purchased from Shanghai Outdo Biotech Co., Ltd. HOrgC120PG05 contains a core/case of normal human organs from thyroid gland, esophagus, stomach, colon, liver, pancreas and lung; as well as 3–5 cores/cases of carcinoma and paired adjacent nontumor tissues from papillary thyroid carcinoma, esophageal squamous cell carcinoma, colon adenocarcinoma, rectal adenocarcinoma, hepatocellular carcinoma, pancreatic ductal adenocarcinoma, lung adenocarcinoma, lung squamous cell carcinoma and clear cell renal cell carcinoma. HLugA150CS03 contains 75 cores/cases of LUAD with matched adjacent normal lung tissue, and HColA150CS02 includes 73 cores/cases of COAD with paired normal colon mucosa. Studies using human tissues were approved by the human ethics Review Committee of Shanghai Outdo Biotech Co., Ltd. (SHYJS-CP-1904010, SHYJS-CP-1901005, and SHYJS-CP-1707009) in agreement with the guidelines set out by the Helsinki Declaration. The SLC25A1 antibody for IHC was obtained from Proteintech (15235-1-AP) used at 1:1000 dilution. The sample staining score was determined by multiplying the score of staining intensity and the percentage score of positive cells. The score of staining intensity was graded as follows: negative (score 0), weak (score 1), medium (score 2), or strong (score 3). The percentage of positive cells was categorized into four grades: 0 (negative), 1 (1–25%), 2 (26–50%), 3 (51–75%), or 4 (76–100%). Tissue scan and image analysis were proceeded using Aperio ImageScope software. GraphPad Prism 8 was used for statistical analysis. Student’s t-test identified the differences between the two groups. Error bars indicated means ± S.D. *P* < 0.05 was considered statistically significant.

### *SLC25A1* survival-associated analysis

Survival information of each TCGA sample, including overall survival (OS) and progression-free interval (PFI), was extracted from the TCGA database [21]. Cox regression analysis was performed to clarify the association of *SLC25A1* expression with the prognosis of various cancers. Forest Plot was used to represent the hazard ratio, 95% confidence interval, and associated *P*-values between *SLC25A1* and survival. The cutoff for *SLC25A1* levels was determined using *P*-value minimization, and the corresponding Kaplan–Meier curves were then plotted within the R environment. Next, the Kaplan–Meier plotter (http://kmplot.com/analysis/) was used to perform survival analyses, including OS and first progression (FP) in lung, gastric, breast, and ovarian cancers [22–25]. Kaplan–Meier survival plots with hazard ratio (HR), 95% confidence intervals (CI) and log-rank *P* were generated by selecting the “auto select best cutoff” function. Additionally, the association between *SLC25A1* expression and survival in pan-cancer was analyzed in PrognoScan (http://dna00bio.kyutech.ac.jp/PrognoScan/index.html) microarray datasets [26]. The threshold was adjusted to a Cox *P*-value < 0.05.

### Association between *SLC25A1* expression and microsatellite instability (MSI), tumor mutational burden (TMB) and immune checkpoint (ICP) genes

The SangerBox online tool (http://sangerbox.com/), a cloud-based platform for TCGA data analysis, was used to explore the relationship between *SLC25A1* expression and MSI, TMB, and ICP genes. The correlation between *SLC25A1* and either MSI or TMB was calculated based on the Spearman method, and the results were visualized using radar plots. Pearson’s rank correlation coefficient was used to describe the association between the *SLC25A1* expression and the expression of 60 common immune checkpoint genes [27].

### Correlation between *SLC25A1* expression and immune infiltration in pan-cancer

The association between *SLC25A1* and immune infiltration was determined and investigated by the TIMER (http://cistrome.org/TIMER/) [28]. The TIMER database contains 10,897 TCGA samples across 32 cancer types to allow the comprehensive assessment of the abundance of immune infiltration. The TIMER algorithm was used to analyze the relationship between *SLC25A1* expression and the abundance of six categories of immune invading cells, including CD4 + T cells, CD8 + T cells, neutrophils, myeloid dendritic cells, macrophage, and B cells. In addition, the relationship between *SLC25A1* expression and the immune score or matrix score was analyzed using the Stromalscore, Immunescore, and ESTIMATE score using SangerBox online tool.

### Functional enrichment analysis of SLC25A1-related genes and proteins

Protein–protein interaction (PPI) network of SLC25A1 was first constructed using GeneMANIA (http://www.genemania.org), which is a helpful web tool for providing PPI networks, generating hypotheses about gene function, and detecting genes with similar functions [29]. The STRING website (https://string-db.org/) was also used to investigate SLC25A1-binding proteins [30]. The main parameters were set as follows: organism as “Homo sapiens”, network type as “full network”, meaning of network edges as “evidence”, active interaction sources as “textmining, experiments, databases”, minimum required interaction score as “Low confidence (0.150)”, and max number of interactors to show as ‘no more than 50 interactors’. Then, the top 100 *SLC25A1*-related genes of all TCGA tumors were obtained through the “Similar Gene Detection” module in GEPIA2 [31]. The correlation coefficient (R) and the *P*-value were indicated in the plot. Moreover, the heatmap of the selected genes was gained via the “Gene_Corr” module of TIMER2, and the *P*-value and partial correlation (cor) were calculated by Pearson's correlation test. The Venn diagram was used to display intersections of SLC25A1-binding proteins and related genes based on the above data from GeneMANIA, STRING, and GEPIA2 databases. Finally, the selected genes were combined to perform Gene Ontology (GO) enrichment analysis and Kyoto encyclopedia of genes and genomes (KEGG) pathway analysis using R package (clusterProfiler, org.Hs.eg.db, ggplot2, and enrichplot) under R software v4.0.3 [32, 33]. *P* < 0.05 was considered statistically significant.

### Dependency of tumor cell lines on *SLC25A1*

The dependency of a panel of tumor cell lines on the *SLC25A1* gene was determined by CRISPR gene editing data from the Broad Institute dependency map (DepMap) portal (https://depmap.org/portal/) [34]. The gene dependency and co-dependency analyses were conducted using the “Perturbation Effects” and “Predictability” modules. The “Achilles_gene_effect.csv” “sample_info.csv” file of the DepMap Public 22Q2 dataset was downloaded for the mining. Data were performed with GraPhpad Prism 8 and Microsoft Excel.

### Cell lines and cell culture

The human lung adenocarcinoma cell lines, A549 and H1299, were obtained from the American Type Culture Collection (Manassas, VA, USA). Cells were cultured in standard conditions according to the Repository’s instructions.

### Generation of LUAD cell lines with knockdown of *SLC25A1*

Small interfering RNA (siRNA) targeting human *SLC25A1* with targeting sequence: 5′- GGAGATTGTGCGGGAACAA-3′ was obtained from Ruibo Company. Lipofectamine 2000 (Invitrogen, 11668) was used to transfect siRNA or plasmids into cells at 70% confluence, according to the manufacturer’s instructions.

### Quantitative real-time PCR

Total RNAs were extracted using TRIzol reagent (Thermo Fisher) and reversely transcribed into cDNA using a cDNA synthesis kit (Marligen Biosciences) according to the manufacturer’s instructions. The Power SYBR Green PCR Master Mix (Takara) was used for quantitative real-time PCR (qRT-PCR). The sequences of the primers were: *SLC25A1*, forward: 5ʹ-CCCCATGGAGACCATCAAG-3ʹ, reverse: 5ʹ-CCTGGTACGTCCCCTTCAG-3ʹ; *ACTB*, forward: 5ʹ-GGCTGTATTCCCCTCCATCG-3ʹ, reverse: 5ʹ-CCAGTTGGTAACAATGCCATGT-3ʹ. The relative gene expression for each sample was normalized to that of *ACTB*. An independent Student’s t test identified the differences between the groups. *P* < 0.05 was considered statistically significant.

### Western blot

Collected cells were homogenized in lysis buffer (5% SDS, 10 mM EDTA, 50 mM NaCl, 10 mM Tris–HCl). Protein concentrations were measured using pierce BCA protein assay (Thermo Fisher, Rockford, IL, USA). Proteins were run on a standard SDS-PAGE and transferred to PVDF membranes. Membranes were blocked in 5% milk, incubated with primary antibody at a concentration of 1: 1000, then incubated with secondary antibody at a concentration of 1: 10 000 and read using ECL detection system (Bio-Rad, Richmond, CA). The anti-SLC25A1 (15235-1-AP) and anti- alpha-tubulin (11224-1-AP) were purchased from Proteintech.

### MTS and colony formation assays

For the MTS assay, cells were seeded in a 96-well plate at a density of 2000 cells per well. Cell viability was determined using a MTS assay system (Promega, Madison, WI, USA) every day for 4 days, following the manufacturer’s instructions.For the colony formation assay, 1000 cells were seeded and incubated in 6-well plates. After 2 weeks of culture, the cells were fixed with icecold methanol, stained with crystal violet, imaged and quantified using AlphaImager HP system (Alpha Innotech). Data were presented as mean ± SD. The statistical significance of differences was determined using the Student's t-test. *P-*value < 0.05 was regarded as statistically significant.

## Results

### SLC25A1 expression in pan-cancer

**Fig. 1:**
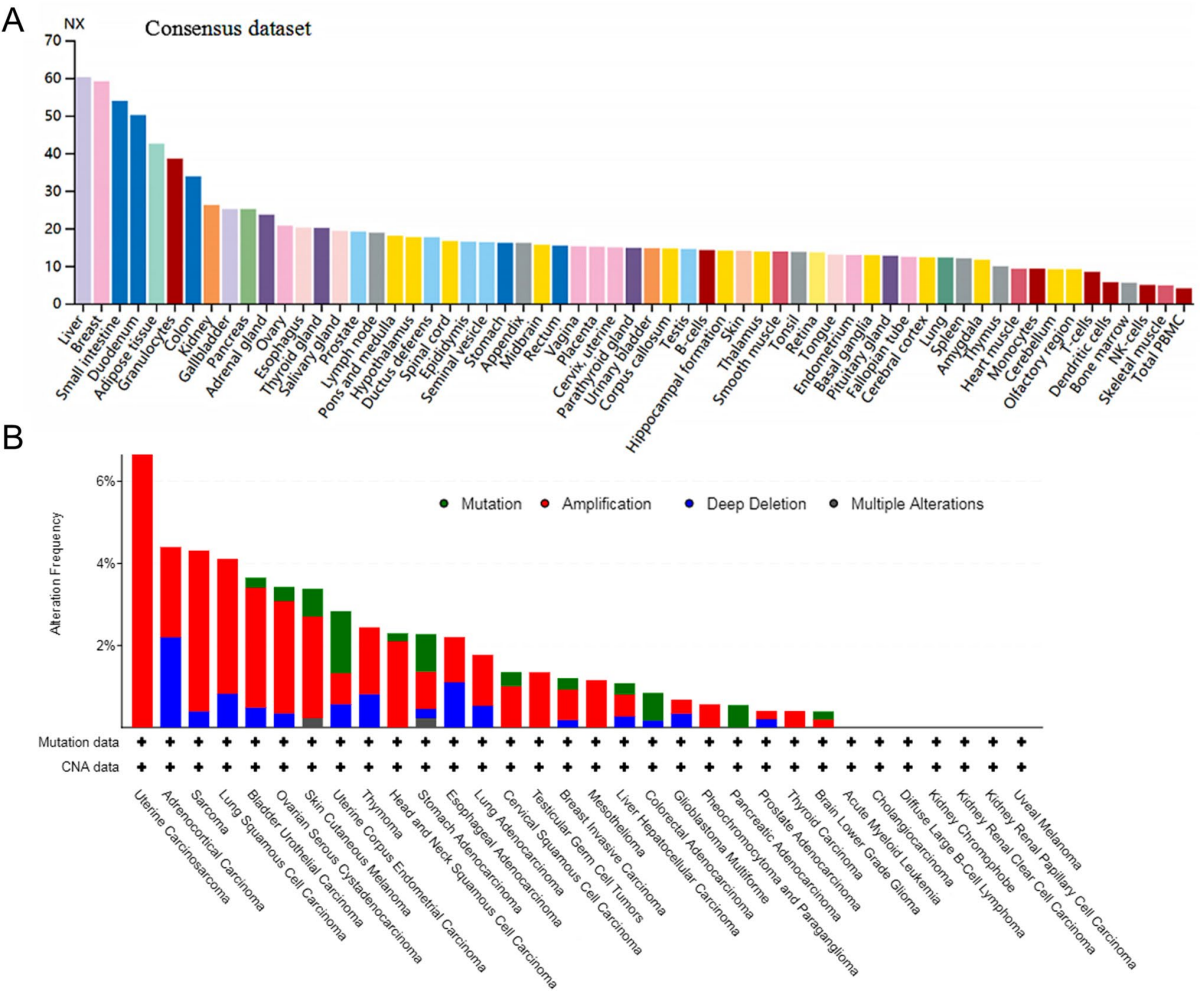
The expression level of *SLC25A1* in normal and tumor tissues. **A** *SLC25A1* mRNA expression in normal tissues based on human protein atlas (HPA) database. **B** Alteration frequency of *SLC25A1* across different cancer types in cBioPortal

We first checked the *SLC25A1* expression in normal tissues using the HPA database. As shown in Fig. 1A, *SLC25A1* mRNA expression was prominent in the liver, breast, small intestine, duodenum, and adipose tissue, all of which have a high content of lipids. We next used cBioPortal to explore genetic aberrations of *SLC25A1* based on mutation, fusion, amplification, deep deletion, and multiple alternations across 32 types of cancer. The results revealed that gene amplification accounted for the most common alteration, while gene mutation rates were generally low (Fig. 1B). Uterine carcinosarcoma (UCS), sarcoma (SARC), lung squamous cell carcinoma (LUSC), bladder urothelial carcinoma (BLCA), and ovarian serous cystadenocarcinoma (OV) had the highest amplification frequencies, i.e., 7.02, 3.92, 3.29, 2.92 and 2.74%, respectively. Therefore, we mainly focused on exploring the expression level and significance of SLC25A1 across human pan-cancers in the following analysis.

**Fig. 2:**
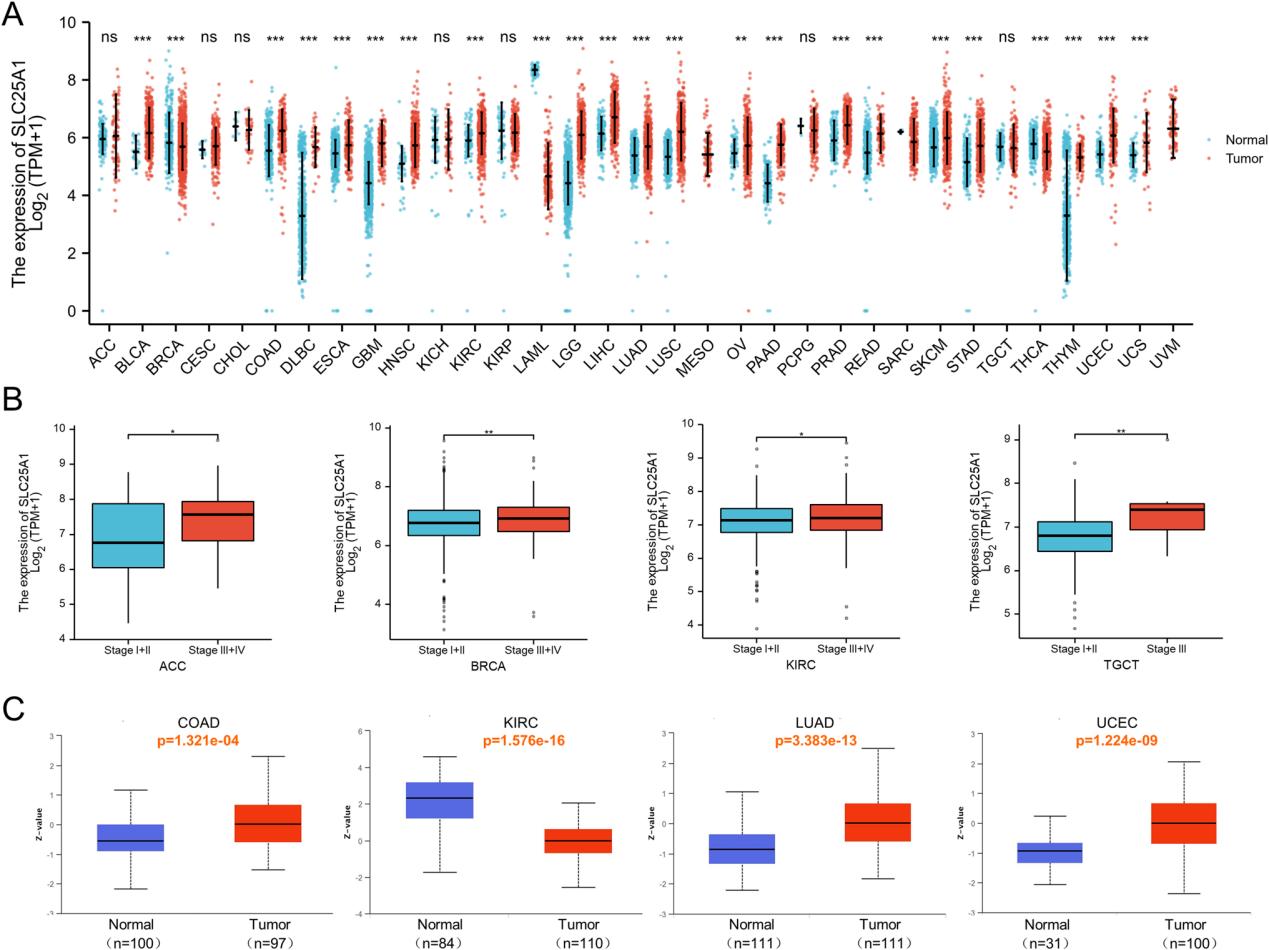
SLC25A1 expression in pan-cancer and different pathological stages. **A** The mRNA expression levels of *SLC25A1* in different types of cancers and normal tissues from the GTEx and TCGA databases. **B** The mRNA levels of *SLC25A1* in different pathological stages (stage I/II and stage III/IV) of ACC, BRCA, KIRC, and TGCT from the TCGA. **C** The protein expression of SLC25A1 in four cancers (COAD, KIRC, LUAD, and UCEC) and normal tissues from the UALCAN database. *P < 0.05, **P < 0.01, ***P < 0.001

The expression of *SLC25A1* mRNA between normal tissues and tumors in pan-cancer was investigated by combining GTEx and TCGA data (Fig. 2A). The results showed that compared with normal tissues, the *SLC25A1* expression in 20 of the 33 cancer types (BLCA, COAD, DLBC, ESCA, GBM, HNSC, KIRC, LGG, LIHC, LUAD, LUSC, PAAD, PRAD, READ, SKCM, STAD, THYM, UCEC, USC, and OV) was significantly higher. In comparison, its expression in 3 types (BRCA, LAML, and THCA) was lower. Meanwhile, the area under the curve (AUC) values for ROC analysis of *SLC25A1* mRNA expression was performed in each cancer (Supplementary Fig. 1). The results indicated that *SLC25A1* had certain accuracy (AUC > 0.7) in predicting 16 cancer types, including BLCA, COAD, DLBC, GBM, HNSC, LAML, LGG, LIHC, LUSC, PAAD, PRAD, READ, STAD, THYM, UCEC, and UCS. Among them, *SLC25A1* had high accuracy (AUC > 0.9) in predicting GBM, LAML, LGG, and PAAD. We also explored the correlation between *SLC25A1* expression and the pathological stages of cancers using TCGA data (Fig. 2B). Its expression was observed significantly increase in the advanced stage (stage III/ IV) relative to early-stage (stage I/ II) in ACC, BRCA, KIRC, and TGCT, suggesting a role for *SLC25A1* in tumor suppression. Moreover, UALCAN bioinformatic analysis was used to analyze SLC25A1 protein expression comprehensively (Fig. 2C). There was elevated expression in the tumor tissues of COAD, LUAD, and UCEC (P < 0.001). Noteworthy, SLC25A1 protein expression in KIRC was lower than that in the normal tissues (P < 0.001), which was in contrast to the mRNA expression.

**Fig. 3:**
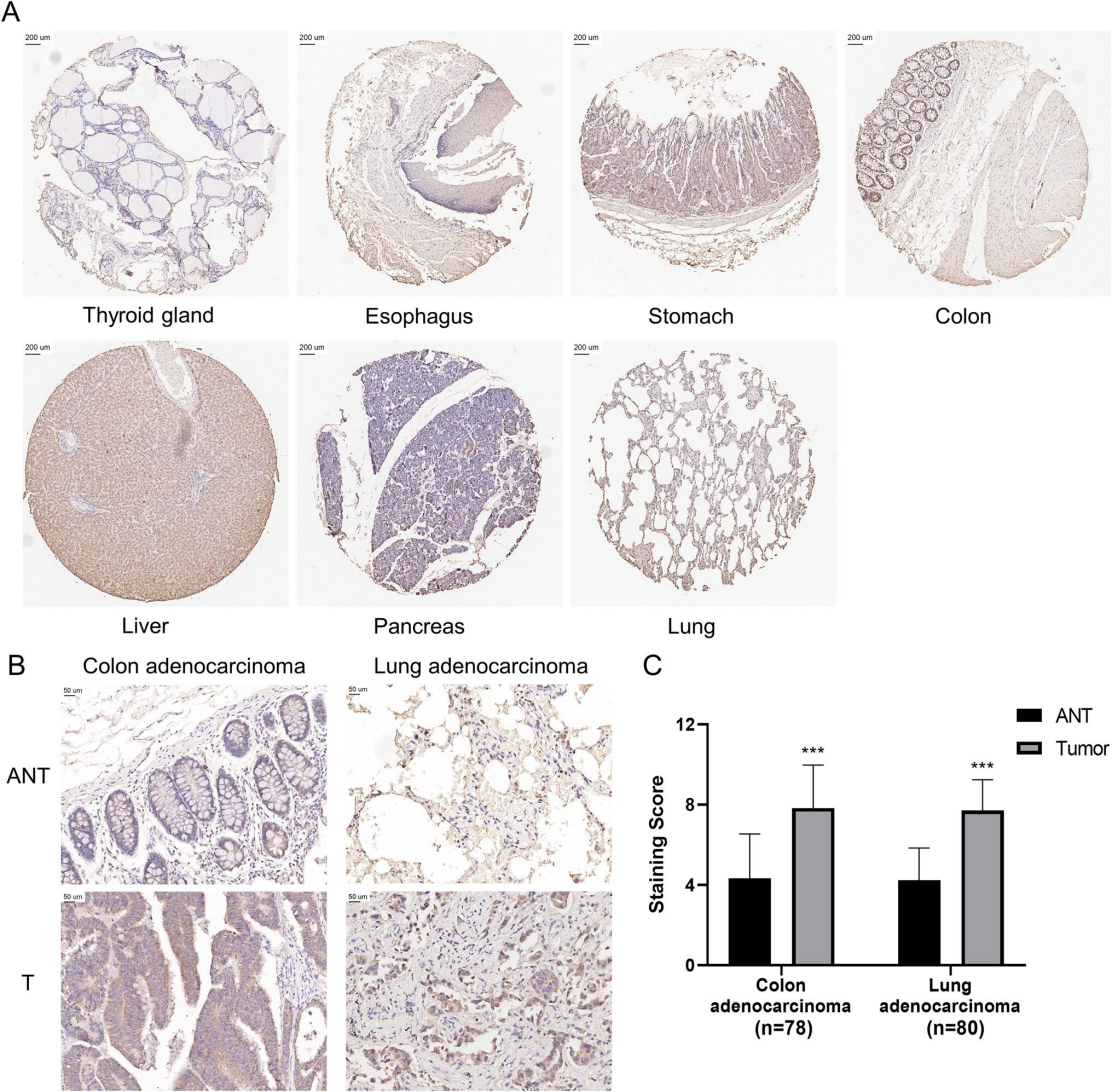
Immunohistochemical analysis of the expression of SLC25A1 in normal and tumor tissues. **A** The protein expression of SLC25A1 in seven normal tissues (thyroid gland, esophagus, stomach, colon, liver, pancreas, and lung). **B** Typical results of one pair of samples in COAD and LUAD. **C** Statistical analysis of the staining score. Bars, means ± SD. *P < 0.05, ** P < 0.01

To further study the expression of SLC25A1, we performed immunohistochemical staining (IHC) in tissue microarrays containing several kinds of human normal tissues and cancers. High SLC25A1 protein expression was observed in normal liver tissue (Fig. 3A). This result was consistent with the mRNA expression data from the HPA database. Furthermore, the IHC showed that the expression levels of SLC25A1 in COAD and LUAD were significantly increased compared with adjacent normal tissues (Fig. 3B, C).

### *SLC25A1* prognostic value in pan-cancer

**Fig. 4:**
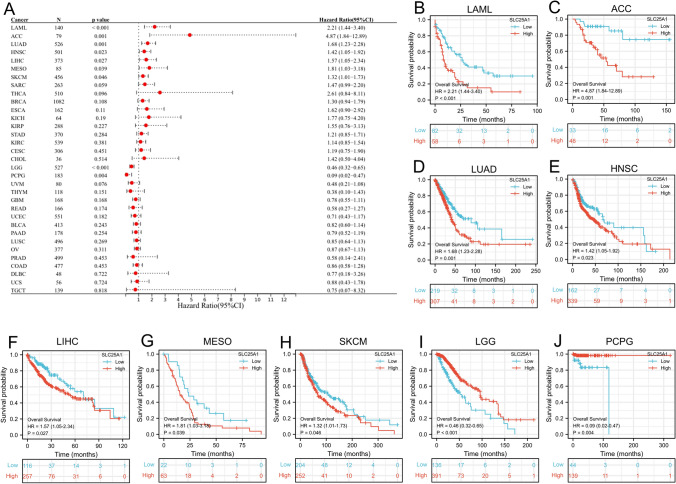
Correlation between *SLC25A1* expression and OS from TCGA database. **A** Analysis of the relationship between *SLC25A1* expression and OS in 33 cancer types using univariate Cox regression and forest plot. The expression of SLC25A1 was related to the survival rate of LAML (**B**), ACC (**C**), LUAD (**D**), HNSC (**E**), LIHC (**F**), MESO (**G**), SKCM (**H**), LGG (**I**) and PCPG (**J**). OS, overall survival

We examined the prognostic values of *SLC25A1* across 33 cancer types in the TCGA database (Fig. 4A). Our study revealed that increased expression of *SLC25A1* was significantly related with worse OS in LAML (HR 2.21 [95% CI 1.44–3.40], *P* < 0.001), ACC (HR 4.87 [95% CI 1.84–12.89], *P* = 0.001), LUAD (HR 1.68 [95% CI 1.23–2.28], p = 0.001), HNSC (HR 1.42 [95% CI 1.05–1.92], *P* = 0.023), LIHC (HR 1.57 [95% CI 1.05–2.34], *P* = 0.027), MESO (HR 1.81 [95% CI 1.03–3.18], *P* = 0.039) and SKCM (HR 1.32 [95% CI 1.01–1.73], *P* = 0.046) (Fig. 4B–H). Conversely, high *SLC25A1* expression was correlated to better OS in LGG (HR 0.46 [95% CI 0.32–0.65], *P* < 0.001) and PCPG (HR 0.09 [95% CI 0.02–0.47], *P* = 0.004) (Fig. 4I-J). Additionally, PFI analyses indicated that high *SLC25A1* expression predicted unfavorable outcomes in individuals with ACC, ESCA, HNSC, KIRC, PRAD, and TGCT, while the opposite trend was observed in individuals with LGG (Supplementary Fig. 2).

**Fig. 5:**
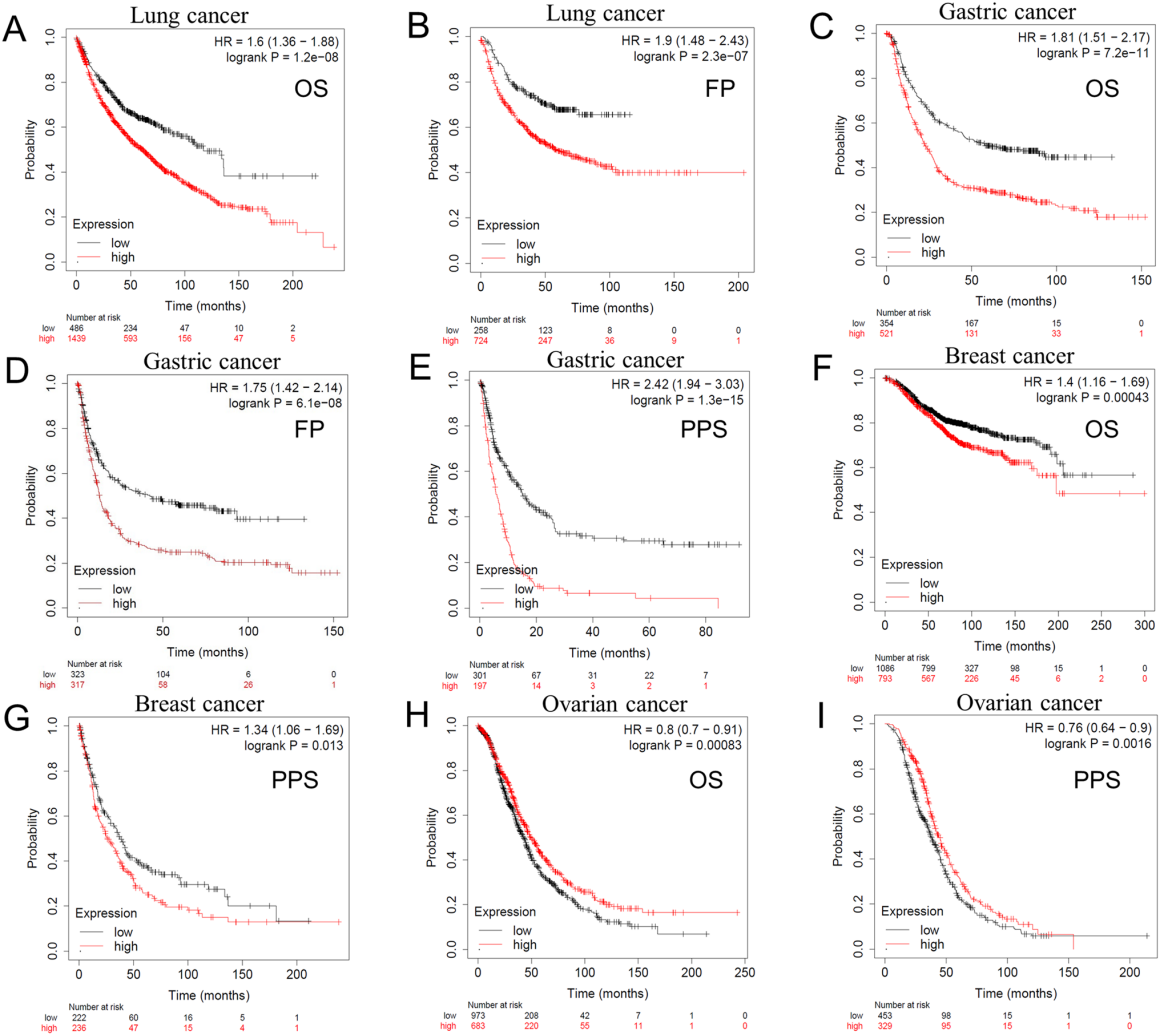
Correlation between *SLC25A1* expression and prognosis of different types of cancer in Kaplan–Meier Plotter database. OS (**A**) and FP (**B**) of lung cancer; OS (**C**), FP (**D**) and PPS (**E**) of gastric cancer; OS (**F**) and PPS (**G**) of breast cancer; OS (**H**) and PPS (**I**) of ovarian cancer. OS, overall survival; FP, first progression; PPS, post-progression survival

Then, the relationship between *SLC25A1* expression and tumor prognosis was checked based on GEO datasets. Using the gene chip data in Kaplan–Meier Plotter, we found that SLC25A1 was a high-risk gene in lung cancer (OS: HR = 1.6, log-rank *P* = 1.2e-08; FP: HR = 1.9, log-rank *P* = 2.3e-07), gastric cancer (OS: HR = 1.81, log-rank *P* = 7.2e-11; FP: HR = 1.75, log-rank *P* = 6.1e-08; PPS: HR = 2.42, log-rank *P* = 1.31e-15) and breast cancer (OS: HR = 1.4, log-rank *P* = 0.00043; PPS: HR = 1.34, log-rank *P* = 0.013) (Fig. 5A–G), but a gene of low risk in ovarian cancer (OS: HR = 0.8, log-rank *P* = 0.00083; PPS: HR = 0.76, log-rank *P* = 0.0016) (Fig. 5H, I). Intriguingly, further analysis showed that *SLC25A1* was associated with a poor prognosis in lung adenocarcinoma, but was not correlated to OS or FP in lung squamous cell carcinoma (Supplementary Fig. 3). The impact of *SLC25A1* on survival was also evaluated through PrognoScan. The results are summarized in Supplementary Table 1. Similar to our findings in Kaplan–Meier Plotter and TCGA database, *SLC25A1* played a detrimental role in breast cancer, lung cancer (adenocarcinoma), and skin cancer (melanoma). Meanwhile, *SLC25A1* had a protective role in brain cancer. Notably, patients with ovarian cancer displayed the opposite trend in DUKE-OC and GSE8841. These findings suggested that *SLC25A1* expression is differentially related to the prognosis in pan-cancer and may be a potential prognostic marker in certain types of tumors.

### Relationship between *SLC25A1* expression and MSI, TMB, and immune checkpoint genes in pan-cancer

**Fig. 6:**
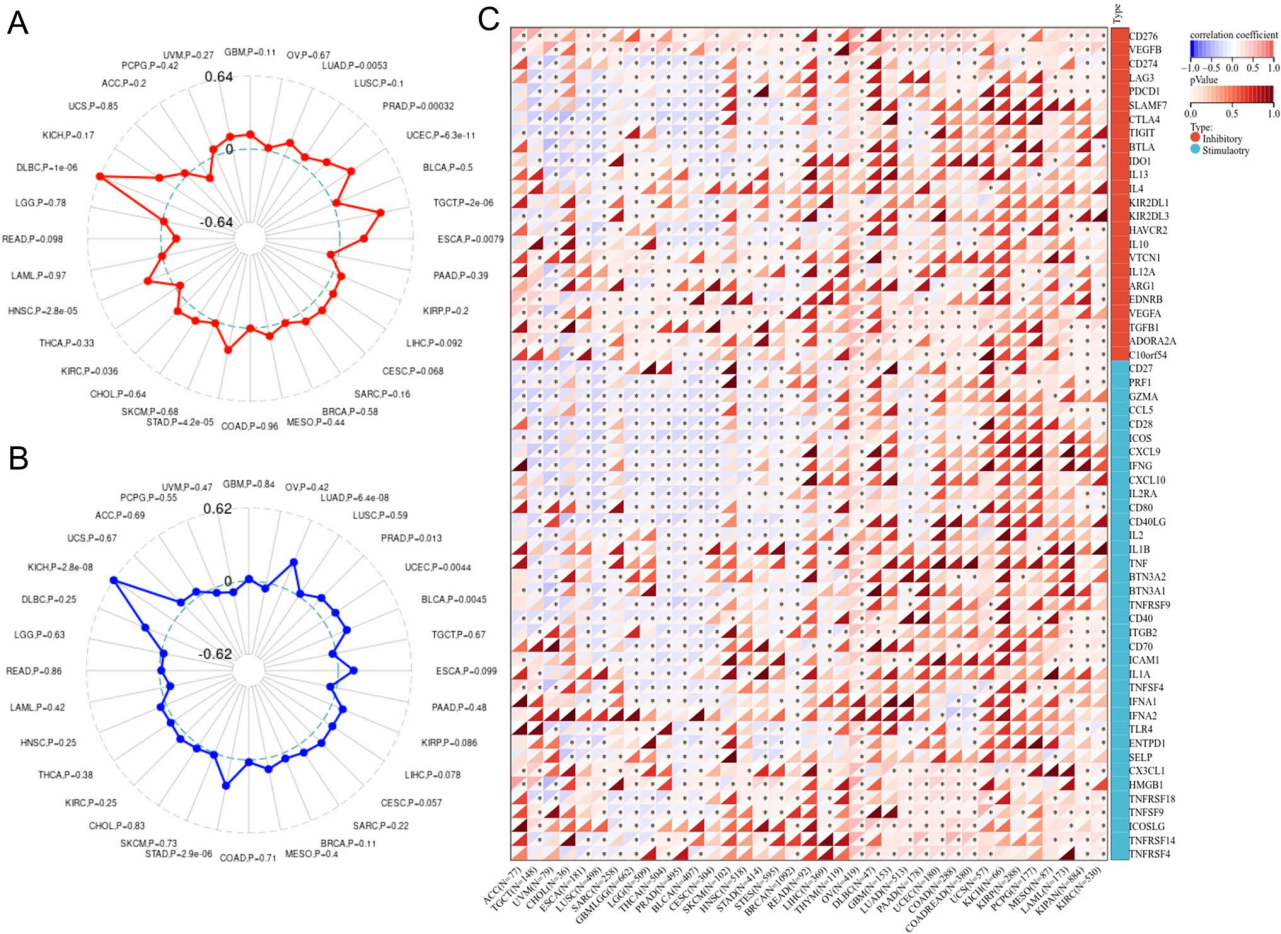
Association between *SLC25A1* expression and microsatellite instability (MSI), tumor mutation burden (TMB), and immune checkpoint genes from TCGA database. **A** Association of *SLC25A1* expression with MSI in different cancers. **B** Association of *SLC25A1* expression with TMB in different cancers. **C** Association of *SLC25A1* expression with immune checkpoint genes in different cancers. * P < 0.05

Microsatellite instability (MSI) and Tumor mutation burden (TMB) were considered to be important biomarkers in predicting the response to ICIs, which are represented by anti–programmed death-1/ligand-1 (PD-1/PD-L1) and anti–cytotoxic T lymphocyte antigen-4 (CTLA-4) inhibitors. Hence, we analyzed the correlation between *SLC25A1* expression and MSI/TMB in diverse cancer types of TCGA. The results revealed that *SLC25A1* was positively associated with the MSI in DLBC (*P* = 1e-06), LUAD (*P* = 0.0053), PRAD (*P* = 0.00032), UCEC (*P* = 6.3e-11), TGCT (*P* = 2e-06), ESCA (*P* = 0.0079), STAD (*P* = 4.2e-05), KIRC (*P* = 0.0036) and HNSC (*P* = 2.8e-05) (Fig. 6A). We further found that SLC25A1 expression was positively correlated with TMB in KICH (*P* = 2.8e-08), LUAD (*P* = 6.4e-08), PRAD (*P* = 0.013), UCEC (*P* = 0.0044), BLCA (*P* = 0.0045) and STAD (*P* = 2.9e-06) (Fig. 6B). Interestingly, no significant negative correlation between MSI/TMB and SLC25A1 expression was observed in all these types of cancer.

Subsequently, we evaluated the associations of *SLC25A1* expression and common immune checkpoint genes. The heat map showed that SLC25A1 exhibited a positive correlation in OV and a negative correlation in LUSC, PRAD and BLCA with more than 40 significant co-expressed immune genes (Fig. 6C). In addition, SLC25A1 expression was positively correlated with *CD276* and *VEGFB* but negatively associated with many known immune checkpoints, including *CD274*, *PDCD1*, and *CTLA-4* in multiple cancers. These results indicated that the relationship between *SLC25A1* and immune checkpoints varies by both tumor type and checkpoint-gene specificity.

### Correlation between *SLC25A1* expression and immune infiltration across cancers

**Fig. 7:**
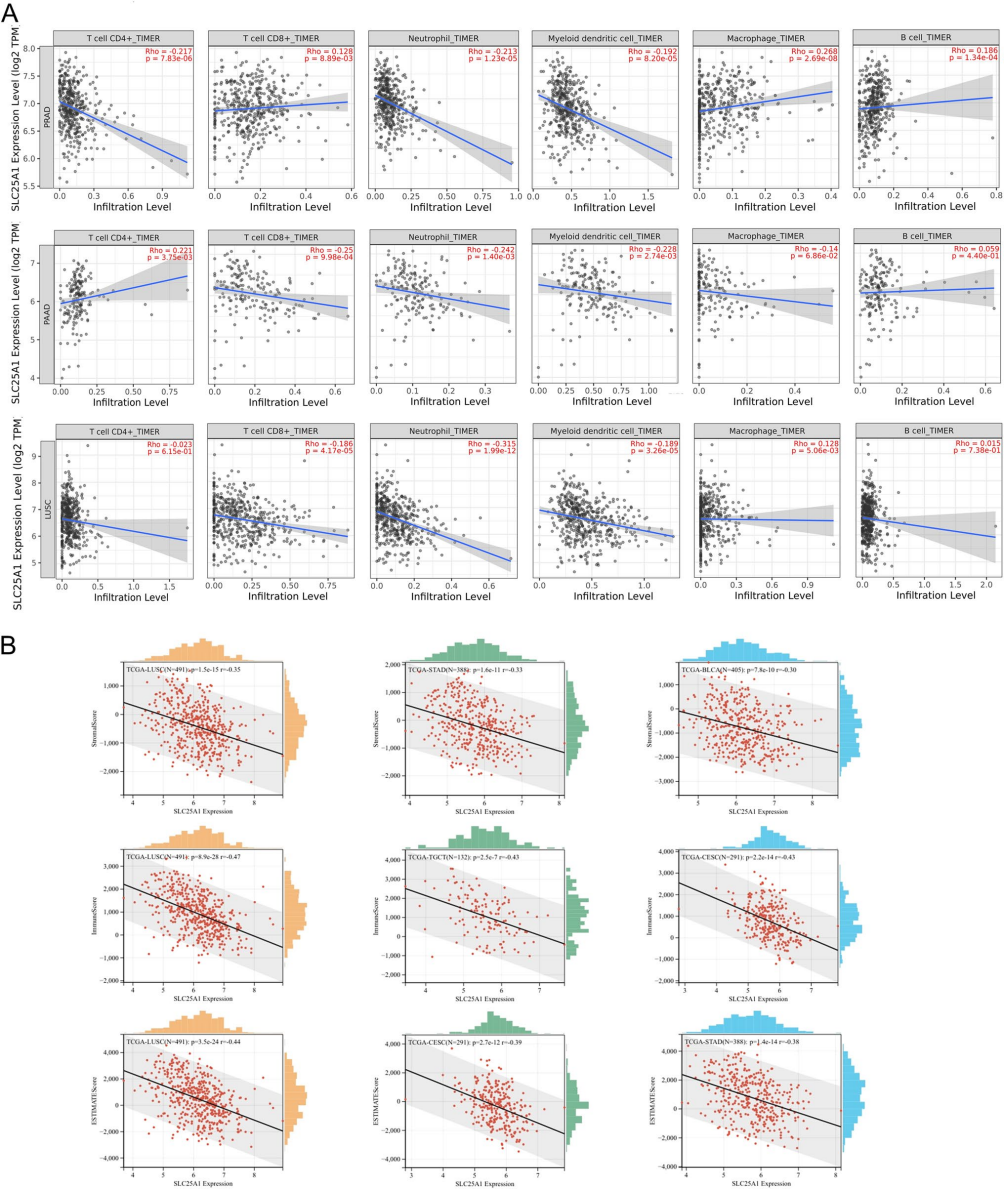
Top 3 cancers related to immune infiltration, Stromalscore, Immunescore, and ESTIMATE score. **A** Association between *SLC25A1* expression and the level of immune infiltration in PRAD, PAAD, and LUSC. **B** Top 3 cancers related with Stromalscore, Immunescore, and ESTIMATE score derived by ESTIMATE algorithm

Tumor-infiltrating immune cells are an essential part of the tumor microenvironment (TME) and have been identified as potential biomarkers for predicting prognosis and response to treatment in cancer patients. Following that, we searched the relationship between *SLC25A1* expression and infiltration level of 6 immune cell types (CD4 + T cells, CD8 + T cells, neutrophils, myeloid dendritic cells, macrophage, and B cells) in pan-cancer from the TIMER database. The top three tumors where *SLC25A1* expression showed the most relevance to immune infiltration levels were PRAD, PAAD, and LUSC (Fig. 7A). Among them, *SLC25A1* expression showed positive relevance with CD8 + T cells (R = 0.128, *P* = 8.89e-03), macrophage (R = 0.268, *P* = 2.69e-08)and B cells (R = 0.186, *P* = 1.34e-04) but negative correlation with CD4 + T cells (R = − 0.217, *P* = 7.83e-06), neutrophils (R = − 0.213, *P* = 1.23e-05) and myeloid dendritic cells (R = − 0.192, *P* = 8.20e-05) in PRAD. In PAAD, *SLC25A1* expression has a significant positive correlation with the immune infiltrating levels of CD4 + T cells (R = 0.221, *P* = 3.75e-03) but negative correlation with the infiltrating levels of CD8 + T cells(R = − 0.25, *P* = 9.98e-04), neutrophils (R = − 0.242, *P* = 1.40e-03) and myeloid dendritic cells (R = − 0.228, *P* = 2.74e-03). In addition, *SLC25A1* expression was significantly and negatively correlated with infiltrating levels of CD8 + T cells (R = − 0.186, *P* = 4.17e-05), neutrophils (R = − 0.315, *P* = 1.99e-12), myeloid dendritic cells (R = − 0.189, *P* = 3.26e-05) and macrophage (R = − 0.128, *P* = 5.06e-03) in LUSC.

The Estimation of Stromal and Immune cells in Malignant Tumor tissues using the Expression data (ESTIMATE) algorithm could help to determine the fraction of stromal and immune cells in the tumor microenvironment. The present study found that *SLC25A1* gene expression was negatively correlated with Stromalscore, Immunescore, and ESTIMATE score (Fig. 7B). The expression of *SLC25A1* was most associated with StromalScore in LUSC (r = − 0.35), STAD (r = − 0.33), and BLCA (r = − 0.30) and significantly negatively correlated with ImmuneScore in LUSC (r = − 0.47), TGCT (r = − 0.43), and CESC (r = − 0.43). Similarly, There were negative correlations between SLC25A1 expression and ESTIMATE score in LUSC (r = − 0.44), CESC (r = − 0.39) and STAD (r = − 0.38). These results indicated that *SLC25A1* expression was closely related to the tumor microenvironment in certain tumor types.

### SLC25A1-related proteins and genes enrichment analysis

**Fig. 8:**
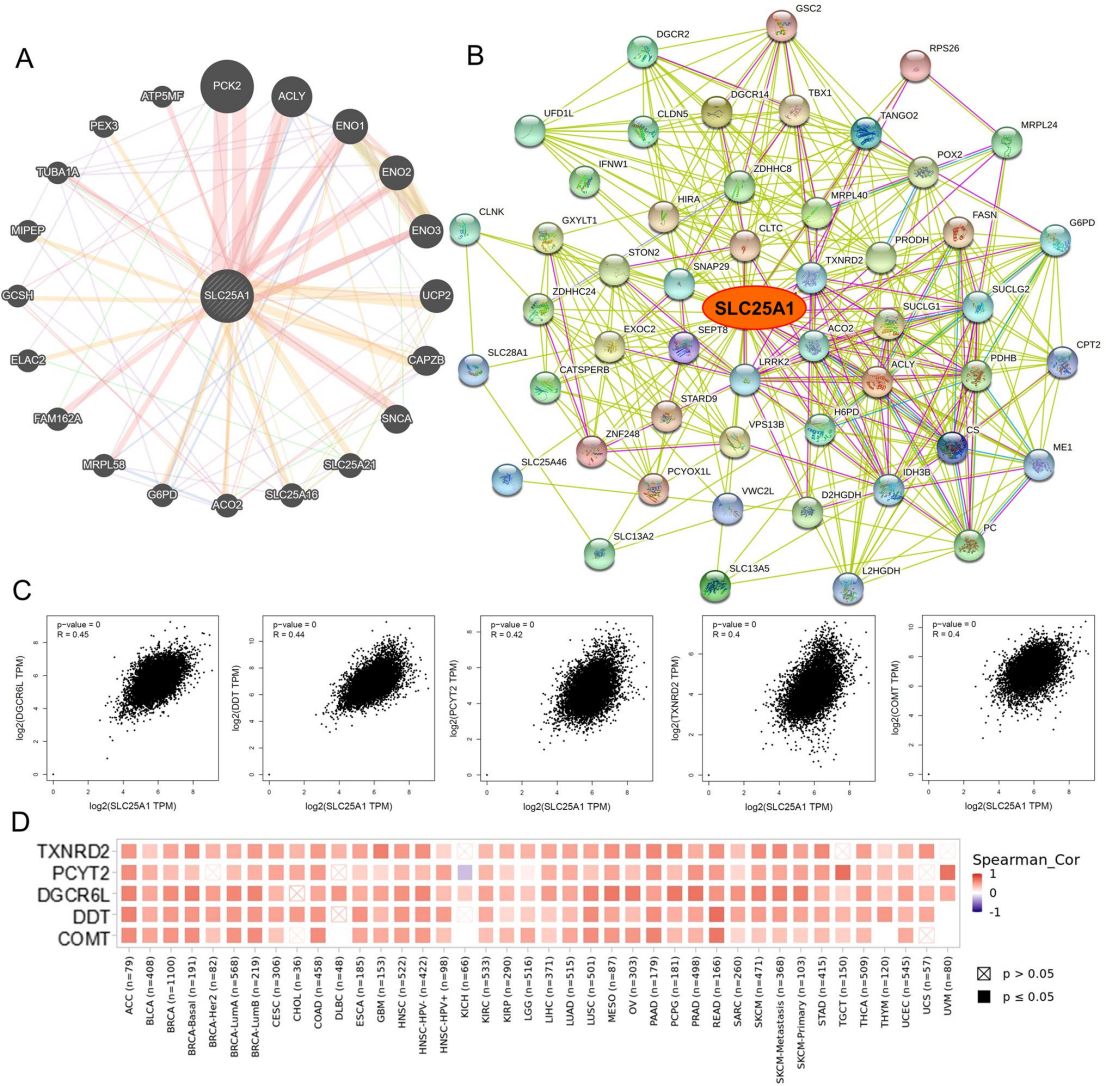
SLC25A1-related protein and genes analysis. **A** Protein–protein interaction (PPI) network of SLC25A1 using GeneMANIA. **B** Protein–protein interaction (PPI) network of SLC25A1 using STRING. **C** The expression correlation between *SLC25A1* and *DGCR6L*, *DDT*, *PCYT2*, *TXNRD2*, *COMT* utilizing GEPIA2. **D** Heatmap of the expression correlation between *SLC25A1* and *DGCR6L*, *DDT*, *PCYT2*, *TXNRD2*, *COMT* utilizing TIMER database

To further uncover the biological significance of SLC25A1 in carcinogenesis at the pan-cancer level, we screened SLC25A1-related proteins and genes and conducted subsequent pathway enrichment analysis. By using GeneMANIA and STRING, we generated the protein–protein interaction network of SLC25A1. Figure 8A from GeneMANIA showed 20 genes most related to SLC25A1 and a PPI network. Furthermore, the STRING database was applied to acquire an interaction network of the top 50 SLC25A1-binding proteins (Fig. 8B). Then, we studied the top 100 genes positively correlated with SLC25A1 expression in pan-cancer of TCGA with the GEPIA2 tool. The scatter plots displayed that *SLC25A1* expression was statistically related to that of the top 5 genes, including *DGCR6L* (R = 0.45), *DDT* (R = 0.44), *PCYT2* (R = 0.42), *TXNRD2* (R = 0.4) and *COMT* (R = 0.4) (Fig. 8C). The heat map from the TIMER database further confirmed a positive relationship between *SLC25A1* expression and the above five genes in most cancer types of TCGA (Fig. 8D).

**Fig. 9:**
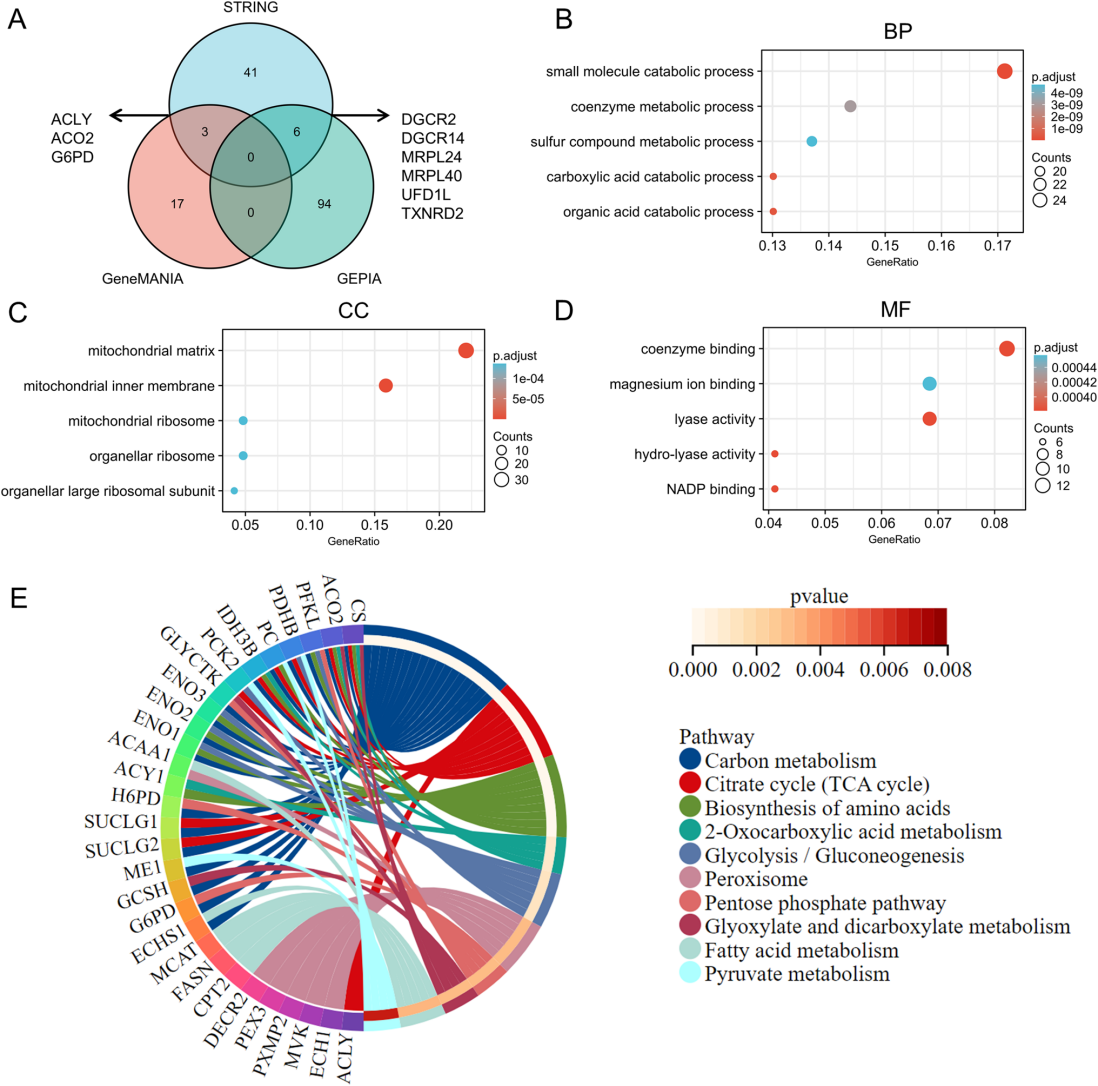
SLC25A1-related genes enrichment analysis. **A** Venn diagram of the SLC25A1- interacted and correlated genes of STRING, GeneMANIA and GEPIA2 database. **B**–**D** GO enrichment analysis of SLC25A1-interacted and correlated genes. **E** KEGG pathway analysis of SLC25A1-interacted and correlated genes

According to the Venn diagram, three common genes of the STRING group and GeneMANIA group were screened out as ACLY, ACO2, and G6PD; while six common members were located in both STRING and GEPIA2 list, including DGCR2, DGCR14, MRPL24, MRPL40, UFD1L and TXNRD2 (Fig. 9A). Combined with the above three databases, we carried out GO and KEGG enrichment analyses. In GO enrichment analysis, these SLC25A1 interacted and correlated genes mainly enriched in the small molecule catabolic process in the biological process (BP), in the mitochondrial matrix in terms of cell component (CC), and in the coenzyme binding in terms of molecular function (MF) (Fig. 9B–D). The KEGG pathway enrichment data further suggested that the selected genes might be involved in the Carbon metabolism, Citrate cycle (TCA cycle), Biosynthesis of amino acids, 2-Oxocarboxylic acid metabolism, Glycolysis/Gluconeogenesis, etc. (Fig. 9E).

### SLC25A1 gene dependency in pan-cancer

In order to comprehensively study SLC25A1 expression on cancer cell growth, we analyzed the vulnerability to CRISPR/Cas9-mediated perturbation of *SLC25A1* in large panels of human cancer cell lines using the DepMap project data (DepMap 22Q2 Public + Score, Chronos). Negative scores of Gene Effect (dependency scores) reflect reduction in cell growth and survival after depletion of a particular gene and the score of -1 corresponds to the median of all common essential genes.

**Fig. 10:**
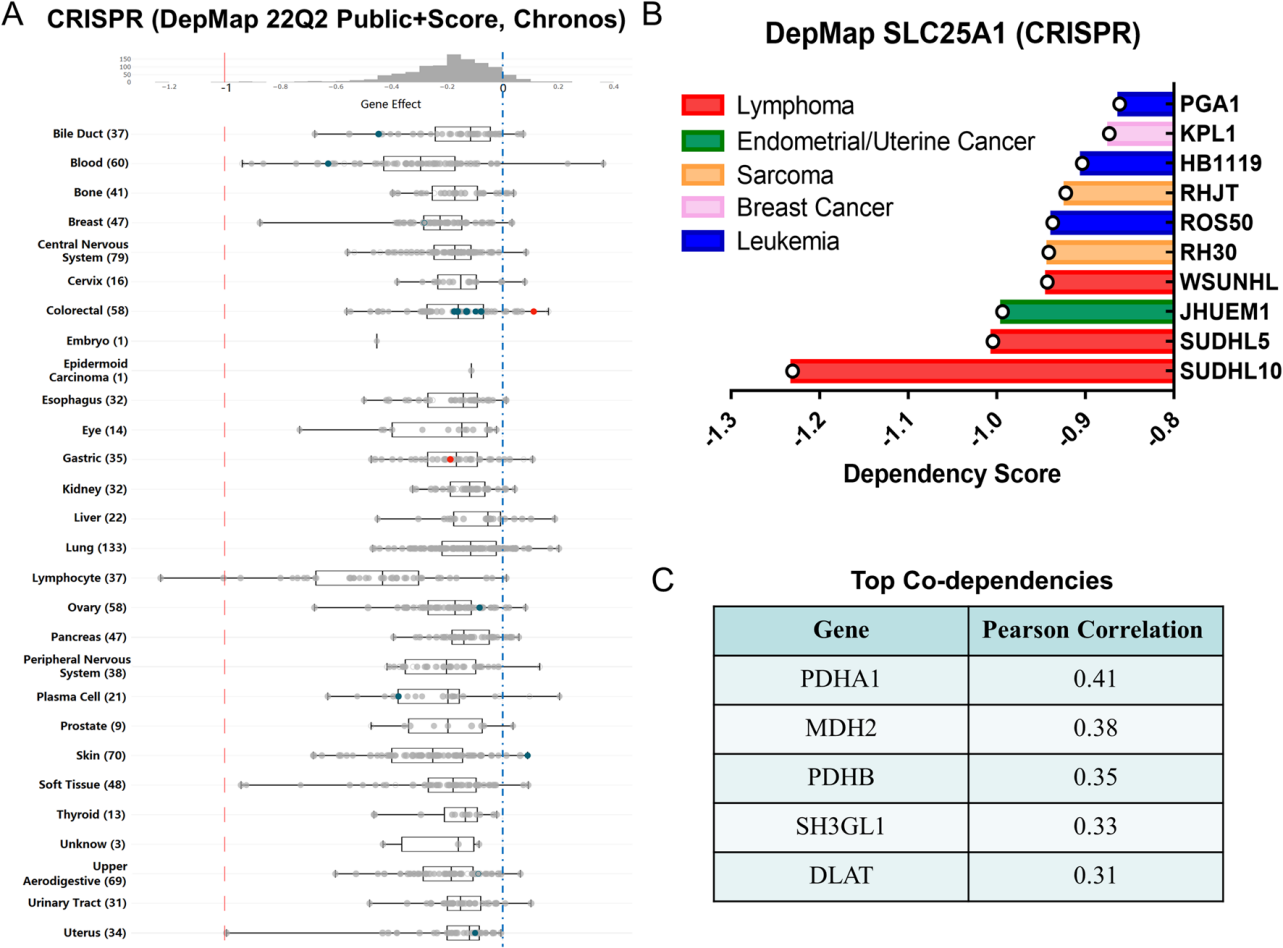
Dependency of cancer cells on *SLC25A1* gene in the DepMap project. **A** Gene effect of CRISPR/Cas9 knockout of *SLC25A1* gene across various human cancer cell lines. More negative scores indicate greater sensitivity to knockout. Each cell line is represented by a circle symbol. **B** Top 10 cancer cell lines sensitive to *SLC25A1* gene knockout. **C** Top 5 Co-Dependencies upon *SLC25A1* deletion by CRISPR/Cas9 in tumor cell lines

Notably, *SLC25A1* knockout showed an inhibitory effect in most cancer cell lines of all cancer types with a median dependency score < 0 (Fig. 10A). The Top10 cell lines sensitive to *SLC25A1* gene knockout were lymphoma cell lines (SUDHL10, SUDHL5, and WSUNHL), endometrial/uterine cancer cell line (JHUEM1), sarcoma cell lines (RH30 and RHJT), leukemia cell lines (ROS50 and HB1119) and breast cancer cell line (KPL1), all of which exhibited a dependency score less than − 0.85 (Fig. 10B). Among them, 2 DLBCL cell lines SUDHL10 and SUDHL5 were the most sensitive cell lines for *SLC25A1*-KO, and both of their dependency scores were lower than—1. Furthermore, we queried the Top Co-dependency Pearson correlations of *SLC25A1*, and *PDHA1*, *MDH2*, *PDHB*, *SH3GL1* and *DLAT* were identified as the top five dependencies (Fig. 10C). Taken together, these findings suggest a potential role of SLC25A1 in clinical applications.

### Effect of *SLC25A1* knockdown on cell proliferation

**Fig. 11:**
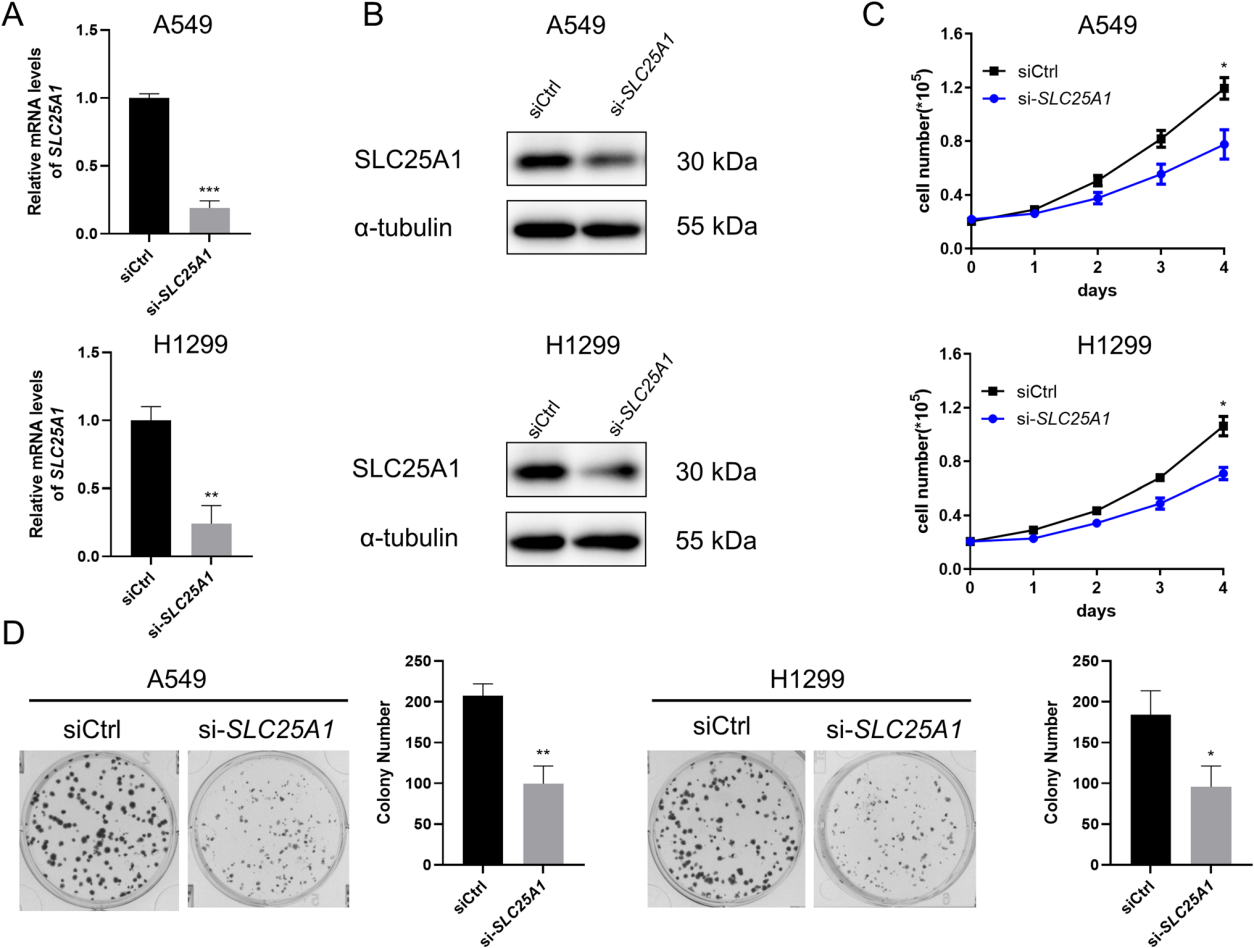
Knockdown of *SLC25A1* attenuated tumor growth in LUAD cells. **A**, **B** Knockdown of *SLC25A1* was confirmed by qRT-PCR and western blot assays in A549 and H1299 cells. **C** Cell viability was determined by MTS assay in A549 and H1299 cells with *SLC25A1* knockdown. **D** Colony formation assay in A549 and H1299 cells with *SLC25A1* knockdown. Bars, means ± SD. *P < 0.05, **P < 0.01, ***P < 0.001

To confirm the function of SLC25A1 in tumor growth, we selected lung adenocarcinoma cell lines to examine the effect of *SLC25A1* knockdown on cell proliferation *in vitro*. We used small interference RNA (siRNA) to knock down the expression levels of *SLC25A1* in A549 and H1299 cell lines (Fig. 11A, B). The MTS assays showed a significant decrease in the viability of *SLC25A1* knockdown cells, compared with the negative control cells (Fig. 11C). The colony formation assays demonstrated that *SLC25A1* knockdown markedly suppressed the proliferation of lung adenocarcinoma cells (Fig. 11D). These observations suggest that SLC25A1 may play a role in LUAD progression.

## Discussion

Metabolic reprogramming has been defined as one of the hallmarks of cancer [35]. Citrate serves as a key metabolite connecting many vital metabolic pathways [36]. As currently the only known mitochondrial citrate transporter, solute carrier family 25 member 1 (SLC25A1) has been proposed to be a metabolic oncogene [6]. Past research has shed light on the critical role of SLC25A1 in the prognosis and therapy of various human cancers [10–12, 37]. In the present study, more than 1 million samples from the cBioPortal, TCGA, GTEx, GEO and DepMap were systematically assessed. The expression profile, prognostic significance, immunological role, enrichment analysis, and dependency analysis were performed in pan-cancer.

The expression of SLC25A1 has been found to be regulated by many factors. The transcriptional activation of SLC25A1 was promoted by p53 mutants and Myc, while it was repressed by the tumor suppressor gene PTEN [6]. Previous studies reported that SLC25A1 expression was enhanced in lung and prostate cancer cell lines under conditions of acute or chronic cycling hypoxia/re-oxygenation stress [7, 13]. Solid tumors frequently generate hypoxic regions by outstripping the vascular supply, and harbor genetic alterations, including TP53 mutation [38, 39]. Consistent with these findings, our study demonstrates that the expression levels of *SLC25A1* were upregulated in most solid tumors, such as COAD, LUAD, and UCEC. Further immunohistochemical tests confirmed that SLC25A1 expression levels were increased in COAD and LUAD compared to corresponding normal tissues, which resembles the findings of previous studies [11, 12]. Among the 33 tumors, three forms of cancer showed downregulation of *SLC25A1* in tumor tissues compared to non-tumor tissues (BRCA, LAML, and THCA). Olga Catalina-Rodriguez et al. showed that expression levels of *SLC25A1* increased in human breast cancer [10]. Our data shows a slight decrease in *SLC25A1* mRNA in BRCA, which extracted and assessed by the GTEx and TCGA database combined. The heterogeneity between studies may be due to differences in analytic methodology, and study sample sizes. It is worth noting that the expression of SLC25A1 was significantly reduced in LAML compared to normal controls. As an hematologic malignancy, LAML have an atypical metabolic profile characterized by a greater reliance on oxidative phosphorylation (OXPHOS) and fatty acid oxidation (FAO) [40]. Intriguingly, our findings indicated that low expression of SLC25A1 is a favorable predictor for LAML patient outcome. The exact role of SLC25A1 in LAML needs further exploration. Earlier literatures showed that high expression of SLC25A1 was correlated with shorter survival time in lung cancer and colorectal cancer patients [12, 13]. We statistically analyzed the prognostic role of SLC25A1 using TCGA, Kaplan–Meier Plotter, and PrognoScan databases. Our study indicates that the prognostic significance of SLC25A1 varies across different cancers. The data suggest that *SLC25A1* mRNA high expression may be an unfavorable prognosis indicator for LAML, ACC, LUAD, HNSC, LIHC, MESO, SKCM, BRCA, and STAD. Among them,, the prognostic implication of *SLC25A1* in LUAD was highly consistent in different databases. Further in vitro verification experiments demonstrated that inhibition of *SLC25A1* could suppress cell proliferation in LUAD cells. Notably, our data also implicates that SLC25A1 might be a favorable prognosis indicator for LGG, PCPG, and OV. This difference may be due to the complexity of the tumor metabolic microenvironment, genetic context, and the intrinsic properties between types of tumors. Future functional studies and independent validation will help to determine whether SLC25A1 can be used as an independent predictive factor in cancers.

Mounting evidence demonstrates that cancer metabolism not only participates in bioenergy, biosynthesis and signaling transduction for tumorigenesis and tumor maintenance but also regulates antitumor immunity and response to immunotherapy through the release of metabolites and modulating the expression of immune-related molecules [41]. Recently, studies have indicated that mitochondria is vital for human immunity and citrate is essential for the activation and immune cells' effector functions, including T cells [36, 42]. However, to our knowledge, no research has evaluated the relationship between SLC25A1 expression and cancer immunology. Among the major predictors for immune checkpoint inhibitors (ICIs), microsatellite instability (MSI) is a well-established predictor, and tumor mutational burden (TMB) is an emerging biomarker [43]. An MSI-high (MSI-H) status or a high TMB generally indicates higher numbers of somatic mutations and mutation-associated neoantigens, which could be more easily recognizable by the immune system and thus more susceptible to immunotherapy [44, 45]. Our study showed that *SLC25A1* expression was positively related to MSI in 9 cancers and TMB in 6 cancers. Among them, LUAD, PRAD, UCEC, and STAD were significantly correlated with MSI and TMB with overlapping relationships.

Furthermore, the study presented a complexity of the associations of *SLC25A1* expression and common immune checkpoint genes. Interestingly, the correlation of *SLC25A1* and *CD276* expression was highly consistent in a variety of tumors. CD276, also known as B7-H3, belongs to the B7 ligand family and is a newly identified immune checkpoint molecule [46]. It has been reported that CD276 is involved in the shaping and development of the tumor microenvironment (TME) by regulating T cell-mediated immune responses and is an attractive target for immunotherapies [47]. Numerous anti-CD276 (B7-H3) preclinical models and clinical trials have indicated its feasibility in clinical application [46, 47]. Therefore, further studies focusing on SLC25A1 expression and tumor immunity may contribute to not only the understanding of existing immunotherapeutic approaches but also the development of novel immune-targeted treatment strategies.

As a crucial component of the TME, tumor-infiltrating immune cells (TIICs) can regulate tumor growth, invasion, metastasis, and the host antitumor immune response by altering the immune status [48]. Increased evidence shows that citrate may play a role in the activation and effector functions of immune cells, such as promoting macrophage activation and modulating T cell proliferation, differentiation and effector functions [36, 49]. The correlation between *SLC25A1* expression and CD4 + T cells, CD8 + T cells, neutrophils, myeloid dendritic cells, macrophage, and B cells was systematically evaluated at the pan-cancer level in this study. *SLC25A1* expression was found to be associated with levels of the six immune cell types mentioned above in multiple cancer types. Especially, *SLC25A1* was consistently negatively associated with levels of neutrophils and myeloid dendritic cells across different cancers including PRAD, PAAD, and LUSC. Neutrophils are a double-edged sword in cancer immunology. Although it facilitates cancer progression by remodeling the extracellular matrix and stimulating angiogenesis, neutrophil-mediated cytotoxicity also leads to tumor cell-killing [50]. Dendritic cells have been reported to be tumor-promoting in some contexts but tumor-suppressive in others [51]. In addition, there was a negative correlation between the expression level of *SLC25A1* and the immune score. These findings suggest that SLC25A1 expression is closely related to the tumor microenvironment, which may affect the tumor cell behavior and influence the prognosis and efficacy of immunotherapy for various cancers.

Several lines of evidence have demonstrated that SLC25A1 enhances cancer growth by promoting mitochondrial oxidative metabolism and de novo lipid synthesis [11, 52]. The increased oxidative phosphorylation (OXPHOS) induced by SLC25A1 supports the resistance and adaptation of cancer cells to radiotherapy, platinum-derived agents, and other metabolic and respiration stress conditions [7]. In addition, citrate exported from the mitochondria through SLC25A1 is a required source of acetyl coenzyme A (acetyl-CoA) for protein and histone acetylation [53]. To better understand the underlying mechanisms of SLC25A1 in cancers, the information on SLC25A1-related proteins and genes for a series of analyses was integrated. ACLY, ACO2, and G6PD were common components of the String and GeneMANIA SLC25A1-PPI networks. ATP citrate lyase (ACLY) is the key enzyme for the de novo synthesis of fatty acids, which converts the conversion of citrate to acetyl-CoA [54]. Mitochondrial aconitase (ACO2) is an essential enzyme that links the TCA cycle to lipid metabolism, catalyzing the interconversion of citrate to isocitrate in the TCA cycle [55]. Glucose-6-phosphate dehydrogenase (G6PD) is the rate-limiting enzyme of the pentose phosphate pathway and the primary actor in PPP-mediated tumor progression [56]. Similar to previous reports, our interaction and enrichment study demonstrated that SLC25A1-related proteins and genes were involved in various metabolic and catabolic processes related to carbon metabolism, citrate cycle (TCA cycle), and fatty acid metabolism. It is noteworthy that SLC25A1 was found to be closely associated with the gene expression of DGCR6L, DDT, PCYT2, TXNRD2 and COMT, and other metabolic pathways with less attention, such as biosynthesis of amino acids, 2-oxocarboxylic acid metabolism and pentose phosphate pathway. These findings have not been investigated previously, further exploration might provide a better understanding of the role of SLC25A1 in cancers.

A growing body of research investigated the effects of the genetic suppression or pharmacologic inhibition of SLC25A1 alone and in combinatorial therapies on cancers *in vitro* and *in vivo*. Catalina-Rodriguez et al. showed that inhibition of SLC25A1 in breast cancer by shRNA or specific inhibitor hampers tumor activity of MBA-MD-231 cells [10]. Jiang et al. reported that *SLC25A1* deletion by CRISPR/Cas9 blunted growth in both monolayer in two-dimensional (2D) culture and spheroids in three-dimensional (3D) culture of lung cancer cells [15]. Yang et al. found *SLC25A1* knockdown suppressed the growth of colorectal cancer cells and induced cell apoptosis [12]. Moreover, the SLC25A1 inhibition enhanced the sensitivity to cisplatin in ovarian carcinoma cells and increased the radiosensitivity of hypoxic lung cancer cells [14]. Additionally, SLC25A1 inhibitor was synthetic lethal with the EGFR inhibitor AZD9291 in non-small cell lung cancer both in vitro and in animal models [11]. Hence, SLC25A1 has been proposed to be a novel therapeutic target in cancer, which spurred the development of selective SLC25A1 inhibitors such as BTA, CTPI-1, and CTPI-2 [6].

In this report, we first analyzed the impacts of *SLC25A1* genetic inhibition by CRISPR/Cas9 at the pan-cancer level via the Dependency Map (DepMap) project. The cancer dependency screens showed that the majority of cancer cell lines display varying degrees of reliance on SLC25A1. Overall, lymphomas were most vulnerable to *SLC25A1* knockout, specifically SUDHL10 and SUDHL5 diffuse large B-cell lymphoma (DLBCL) cell lines. So far, there have been no reports on the role of SLC25A1 in DLBCL. However, recent studies have suggested that certain DLBCL highly depends on OXPHOS and lipid synthesis [57, 58]. Given the critical position of SLC25A1 in mitochondria and lipid metabolism, further research is necessary. Co-dependencies of *SLC25A1* in cancer were also examined using the DepMap data. Four (*PDHA1*, *MDH2*, *PDHB*, and *DLAT*) out of the top five dependencies are genes required for the TCA cycle, which may be attributable to their cooperative function with SLC25A1 in maintaining mitochondrial homeostasis. All of the above suggest that SLC25A1 could be a novel and promising target for cancer therapy.

However, there were potential limitations in our report. Although the SLC25A1 expression across various cancers was investigated using public databases and immunohistochemistry, the sample size of tissue microarray in this study was small, resulting in limited information. Importantly, the prognostic significance, immunological role, and biological effects of SLC25A1 in cancers require follow-up appropriate models in vitro or in vivo and large clinical samples to testify. Deep studies focusing on the functions of SLC25A1 in oncogenesis may facilitate the discovery of new prognostic biomarkers and the development of effective targets for cancer treatment.

## Conclusions

Our analysis is the first comprehensive study of the expression levels, clinical prognosis, as well as its immunological and biological implications of SLC25A1 on a pan-cancer basis. This study shed new light on the role of SLC25A1 in tumor immunity. These findings may contribute to a better understanding of the functional significance of SLC25A1 in tumorigenesis and progression, and provide a valuable resource for personalized immunotherapy and precision medicine.

### Supplementary Information

Supplementary file1 (TIF 1681 KB)

Supplementary file2 (DOCX 594 KB)

## Data Availability

The datasets generated during and/or analyzed during the current study are available from the corresponding author on reasonable request.
