# Supplementary figures and images for: A comprehensive analysis of SLC25A1 expression and its oncogenic role in pan-cancer
Source: Discov Oncol. 2023 Nov 19;14:207. doi: 10.1007/s12672-023-00830-z (PMC10657916; doi:10.1007/s12672-023-00830-z)

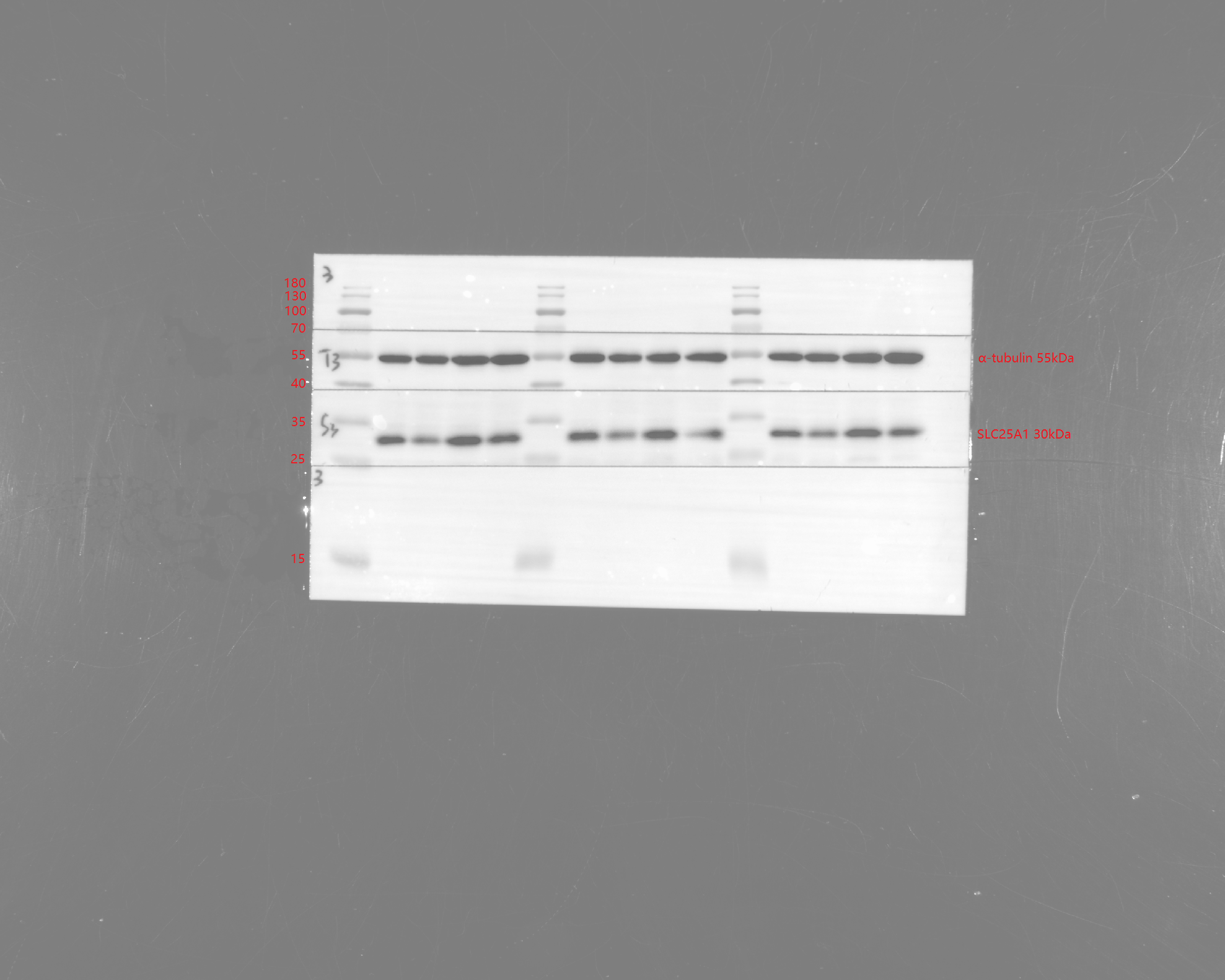

Supplement: Supplementary file 1 — Supplementary file1 (TIF 1681 KB) [file 12672_2023_830_MOESM1_ESM.tif]
