# Supplementary material for: A comprehensive analysis of SLC25A1 expression and its oncogenic role in pan-cancer
Source: Discov Oncol. 2023 Nov 19;14:207. doi: 10.1007/s12672-023-00830-z (PMC10657916; doi:10.1007/s12672-023-00830-z)
Supplement: Supplementary file 2 — Supplementary file2 (DOCX 594 KB) [file 12672_2023_830_MOESM2_ESM.docx]

**Supplementary Table 1. Relation between SLC25A1 expression and patient prognosis of different cancer in PrognoScan database.**

| Cancer type | Dataset | Endpoint | N | Hazard ratio(95%CI) | Cox P |
| --- | --- | --- | --- | --- | --- |
| Brain cancer | GSE4271-GPL96 | Overall Survival | 77 | 0.70 [0.50 - 0.98] | 0.0396255 |
|  | MGH-glioma | Overall Survival | 50 | 0.75 [0.60 - 0.92] | 0.00643142 |
| Breast cancer | GSE9195 | Relapse Free Survival | 77 | 2.38 [1.00 – 5.62] | 0.0487464 |
|  | GSE9893 | Overall Survival | 155 | 1.40 [1.14 - 1.73] | 0.00141275 |
|  | GSE1456-GPL96 | Relapse Free Survival | 159 | 3.12 [1.49 - 6.54] | 0.00250823 |
|  | GSE1456-GPL96 | Disease Specific Survival | 159 | 3.32 [1.40 - 7.90] | 0.00653033 |
|  | GSE3494-GPL96 | Disease Specific Survival | 236 | 2.31 [1.30 - 4.12] | 0.00454712 |
|  | GSE4922-GPL96 | Disease Free Survival | 249 | 2.06 [1.32 - 3.23] | 0.00158794 |
|  | GSE2990 | Distant Metastasis Free Survival | 125 | 2.09 [1.09 - 4.02] | 0.0267711 |
|  | GSE2990 | Relapse Free Survival | 125 | 1.79 [1.08 - 2.97] | 0.0245571 |
|  | GSE7390 | Overall Survival | 198 | 1.86 [1.27 - 2.73] | 0.00151504 |
|  | GSE7390 | Relapse Free Survival | 198 | 1.58 [1.15 - 2.16] | 0.00422566 |
|  | GSE7390 | Distant Metastasis Free Survival | 198 | 1.81 [1.25 - 2.62] | 0.00163706 |
| Lung cancer  (Adenocarcinoma) | GSE31210 | Relapse Free Survival | 204 | 2.95 [1.21 - 7.17] | 0.0172484 |
| Ovarian cancer | DUKE-OC | Overall Survival | 133 | 0.62 [0.47 - 0.82] | 0.000840631 |
|  | GSE8841 | Overall Survival | 81 | 3.78 [1.55 - 9.20] | 0.00344676 |
| Skin cancer  (Melanoma) | GSE19234 | Overall Survival | 38 | 4.24 [1.27 - 14.13] | 0.0186759 |

**Supplemental figure 1**


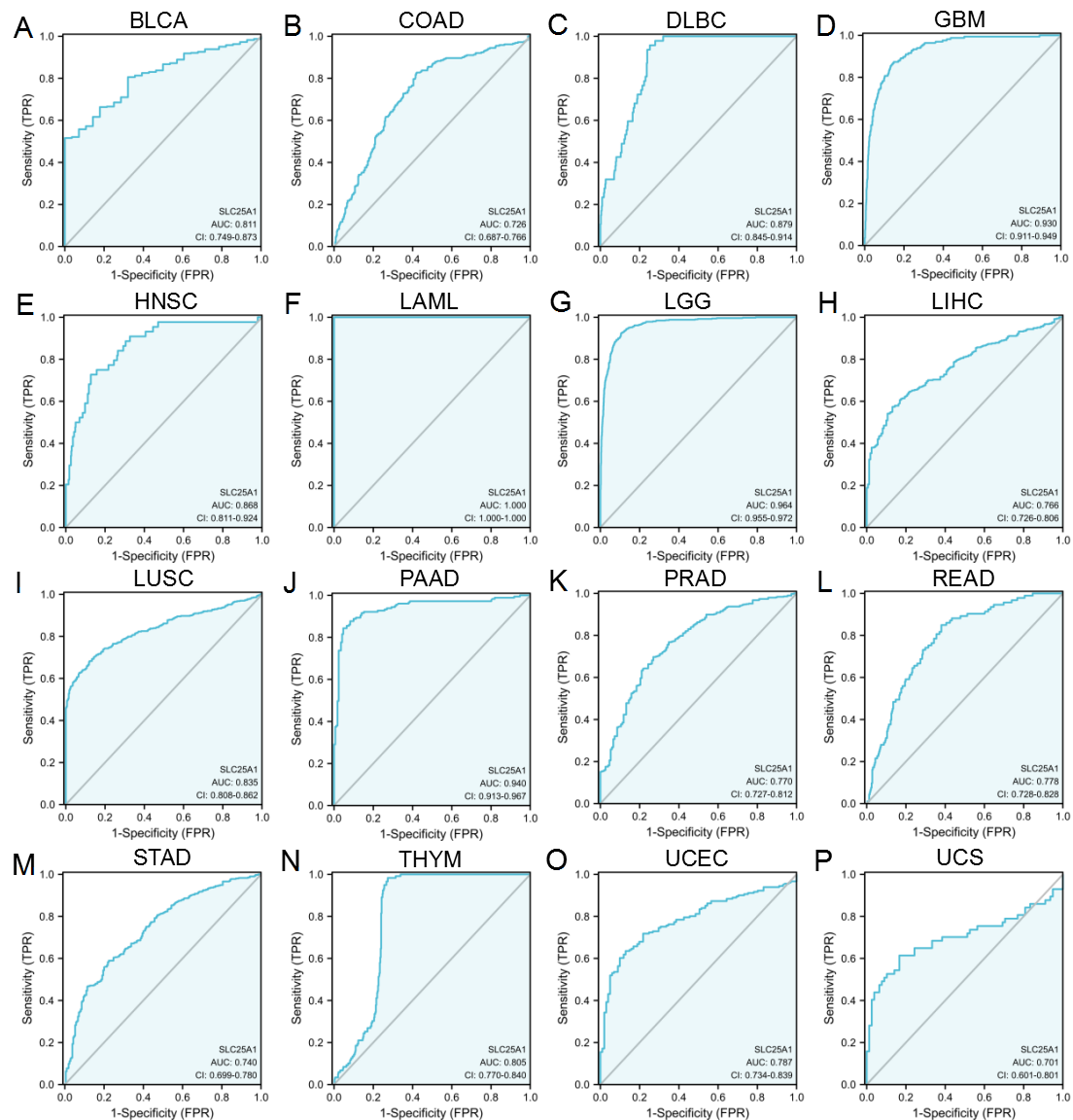


**Supplemental figure 1.** Receiver operating characteristic (ROC) curve for SLC25A1 expression in pan-cancer. (A) BLCA; (B) COAD; (C) DLBC; (D) GBM; (E) HNSC; (F) LAML; (G) LGG; (H) LIHC; (I) LUSC; (J) PAAD; (K) PRAD; (L) READ; (M) STAD; (N) THYM; (O) UCEC; (P) UCS.

**Supplemental figure 2**


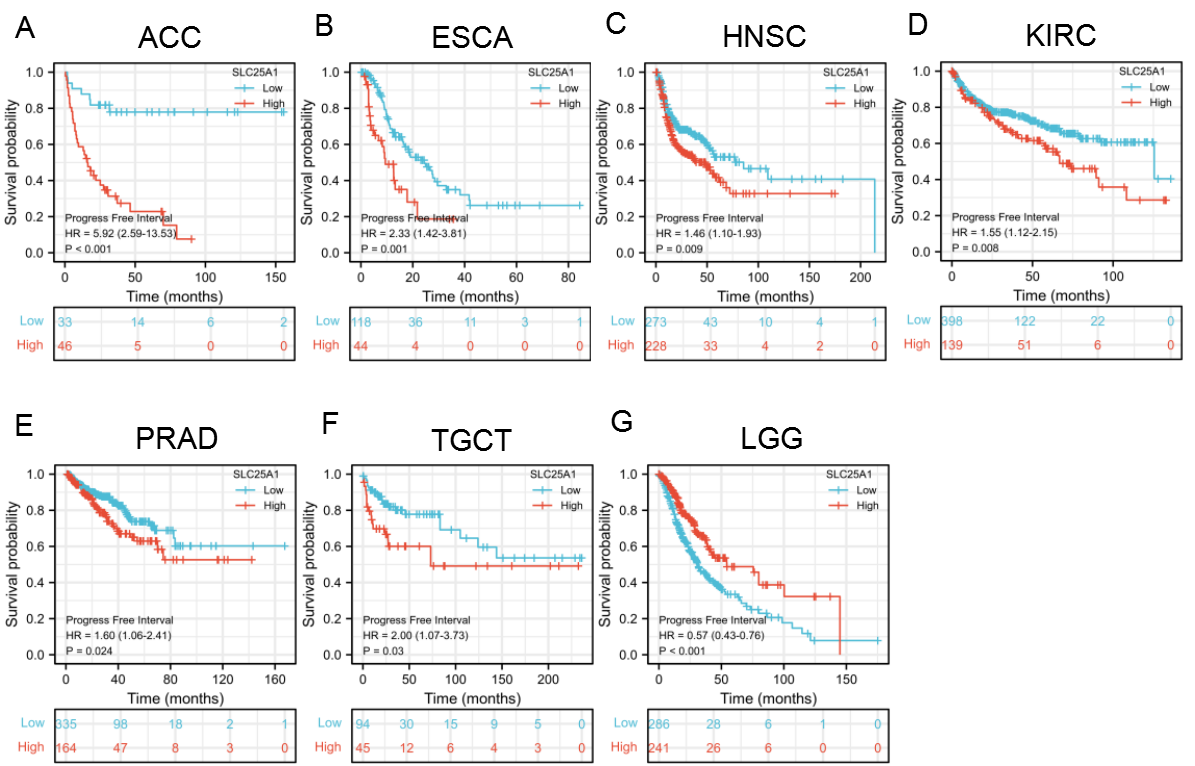


**Supplemental figure 2.** Correlation between SLC25A1 expression with PFI from TCGA database. The expression of SLC25A1 was related to the PFI of ACC (A), ESCA (B), HNSC (C), KIRC (D), PRAD (E), TGCT (F) and LGG (G). PFI, progression-free interval survial.

**Supplemental figure 3**


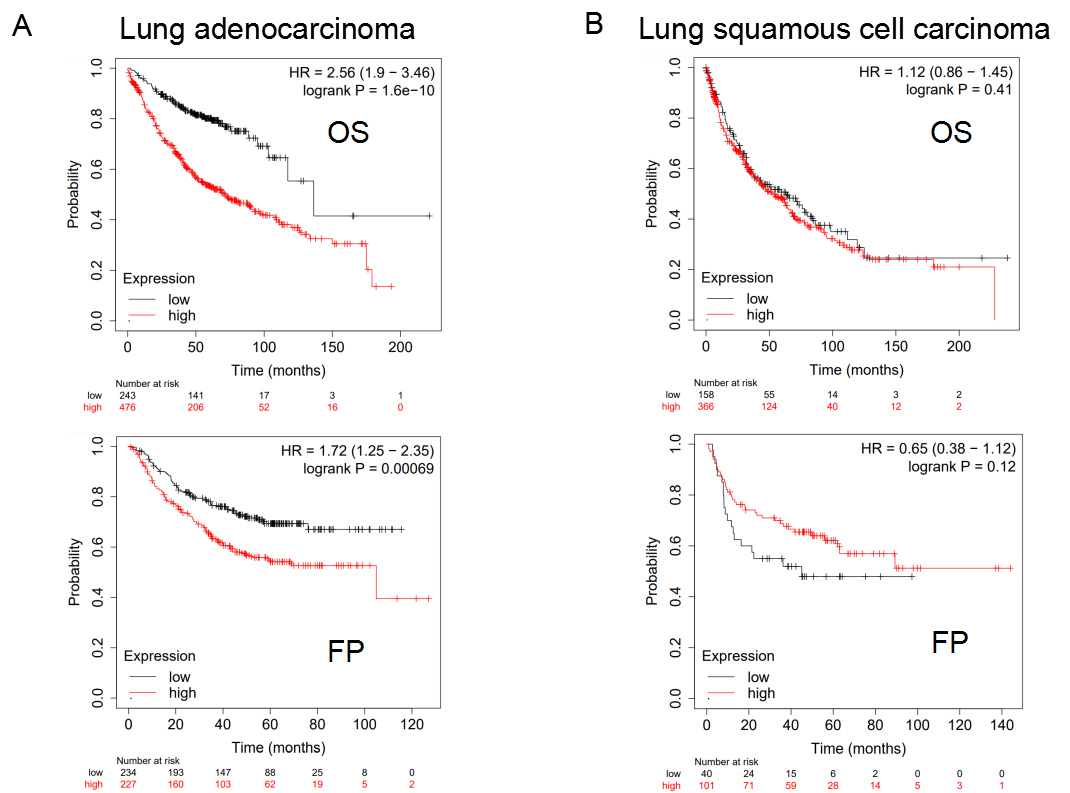


**Supplemental figure 3.** Prognostic values of SLC25A1 expression in different subtypes of lung cancer in Kaplan-Meier Plotter database. (A-B) Correlation between SLC25A1 expression and the prognosis (OS and FP) of lung adenocarcinoma. (C-D) Correlation between SLC25A1 expression and the prognosis (OS and FP) of lung squamous cell carcinoma.
